# Cognitive Function in Peripheral Autonomic Disorders

**DOI:** 10.1371/journal.pone.0085020

**Published:** 2014-01-17

**Authors:** Pietro Guaraldi, Roberto Poda, Giovanna Calandra-Buonaura, Laura Solieri, Luisa Sambati, Roberto Gallassi, Pietro Cortelli

**Affiliations:** 1 IRCCS, Institute of Neurological Sciences of Bologna, Bologna, Italy; 2 Department of Biomedical and Neuromotor Sciences, Alma Mater Studiorum, University of Bologna, Bologna, Italy; Oslo University Hospital, Norway

## Abstract

**Objective:**

aims of the current study were 1) to evaluate global cognitive function in patients with autonomic failure (AF) of peripheral origin and 2) to investigate the effect of a documented fall in blood pressure (BP) fulfilling the criteria for orthostatic hypotension (OH) on cognitive performances.

**Methods:**

we assessed 12 consecutive patients (10 males, 68±7 years old) with pure AF (PAF) or autoimmune autonomic neuropathy (AAN) and 12 age- and gender-matched controls. All patients had no clinical signs of central nervous system involvement and normal brain CT/MRI scan. Cognitive function was assessed on two consecutive days in 3 conditions: on day 1, while sitting, by means of a comprehensive battery of neuropsychological tests; on day 2, while tilted (HUT) and during supine rest (supine) in a randomized manner. BP and heart rate (HR) were continuously recorded non-invasively for the whole duration of the examination.

**Results:**

patients with PAF or AAN displayed a preserved global cognitive function while sitting. However, compared to supine assessment, during HUT patients scored significantly worse during the Trail Making Test A and B, Barrage test, Analogies test, Immediate Visual Memory, Span Forward and Span Backward test. Pathological scores, with regard to Italian normative range values, were observed only during HUT in the Barrage test and in the Analogies test in 3 and 6 patients respectively. On the contrary, in healthy controls, results to neuropsychological tests were not significantly different, during HUT compared to supine rest.

**Conclusions:**

these data demonstrate that patients with PAF and AAN present a normal sitting global cognitive evaluation. However, their executive functions worsen significantly during the orthostatic challenge, possibly because of transient frontal lobes hypoperfusion.

## Introduction

Orthostatic hypotension (OH) is defined as a systolic blood pressure (SBP) fall of at least 20 mmHg or a diastolic blood pressure (DBP) fall of at least 10 mmHg within 3 min of standing or head-up tilt (HUT) to at least 60° [1].

Previous cross-sectional studies reported an association between OH and cognitive decline in various conditions, including central neurodegenerative disorders with autonomic failure (AF) [2]–[4]. However, prospective studies failed to demonstrate that OH was a risk factor for cognitive decline, possibly because of the confounding effects of age, concomitant disorders, medications, cerebrovascular or neurodegenerative processes [4]–[6].

Data on cognitive function in peripheral autonomic disorders, rare conditions characterized by AF without central nervous system (CNS) involvement, are scant. So far, a single retrospective study reported cognitive impairment in 6 out of 14 patients with a longstanding diagnosis of pure AF (PAF) [7]. However, blood pressure (BP) values and cognition were not measured concurrently, patients were tested only while sitting, and 4 patients with cognitive deficits had abnormal CT/MRI scan. A more recent case series on 3 patients with autoimmune autonomic ganglionopathy reported that OH and elevated anti-body titer were associated independently with neuropsychological impairment, which improved, even in the seated normotensive position, after plasmapheresis [8]. In a preliminary non-randomized study based on 10 patients with central and peripheral causes of AF we demonstrated that, despite a normal sitting global cognitive evaluation, our patients presented a significant worsening of global and executive cognitive functions during HUT [9]. However, except for few patients that had some pathological performances in verbal abstract thinking and delayed recall of verbal memory, they scored within the Italian reference range values.

Therefore, due to the paucity of data on cognitive function in peripheral autonomic disorders and since previous studies did not systematically take in consideration the effect of posture on neuropsychological results, we performed the current study aiming at: 1) evaluate global cognitive function in patients with AF of peripheral origin by means of a comprehensive battery of neuropsychological tests performed while sitting; 2) investigate the effect of a documented fall in SBP fulfilling the criteria for OH [1] on cognitive performances, carrying out neuropsychological assessments in a randomized manner while the patients were supine and during HUT.

## Methods

Twelve consecutive patients with a confirmed diagnosis of AF of peripheral origin and twelve age- and gender-matched controls were enrolled in the study. Each participant gave written informed consent before participating to the study, which was approved by the institutional review board of the University of Bologna, Italy.

Patients' inclusion criteria comprised the presence of neurogenic OH [1], absence of clinical signs (parkinsonian, cerebellar or pyramidal) of CNS involvement and normal brain CT/MRI scan (absence of white matter lesions, cortical infarcts, atrophy and hydrocephalus). Of the twelve patients enrolled in the study (10 males, 68±7 years old, mean disease history 12±5 years), 9 had a probable PAF and 3 an autoimmune autonomic neuropathy (AAN) (Table 1 and Table 2). Patients were classified as PAF on the basis of symptoms associated with OH, of results of cardiovascular reflexes and negative cerebrospinal fluid (CSF) examinations [10], [11]. Patients were classified as AAN if presented AF of subacute onset with albuminocytologic dissociation in the CSF [12]. All our AAN patients had negative ganglionic AChR antibodies titers. All patients, except for patient 3, had features of autonomic failure for many years, thus making it most unlikely that this was the autonomic presentation of multiple system atrophy or other central neurodegenerative disorder. All participants were non-smokers and had no additional disorders that might affect cognitive functioning.

**Table 1:** Patients' characteristics and blood pressure and heart rate values while supine and during head-up tilt.

Pt	Sex	Age	Diagnosis	BMI	Dis Hx	Edu	Supine			HUT			Delta (Δ)		
							SBP	DBP	HR	SBP	DBP	HR	SBP	DBP	HR
**1**	M	63	PAF	29.4	14	8	154	80	49	97	59	46	−57	−21	−3
**2**	M	56	AAN	28.4	10	17	128	90	68	79	54	117	−49	−36	49
**3**	M	77	PAF	22.9	10	13	169	76	78	116	51	83	−53	−25	5
**4**	M	70	AAN	24.6	3	5	156	108	59	82	68	54	−74	−40	−5
**5**	M	70	PAF	30.7	14	5	152	104	63	105	75	64	−47	−29	1
**6**	M	67	PAF	30.4	21	8	160	108	59	114	86	84	−46	−22	25
**7**	M	70	PAF	24.1	12	8	168	117	75	85	61	78	−83	−56	3
**8**	F	76	PAF	31.6	14	5	147	107	76	91	61	79	−56	−46	3
**9**	M	58	AAN	26.5	10	17	131	95	70	106	80	80	−25	−15	10
**10**	M	63	PAF	24.1	20	5	182	108	64	126	84	65	−56	−24	1
**11**	F	75	PAF	31.3	10	3	127	95	106	61	52	104	−66	−43	−2
**12**	M	66	PAF	21	11	8	119	78	79	94	68	82	−25	−10	3

Age (years); AAN: Autoimmune autonomic neuropathy; BMI: body mass index; DBP diastolic blood pressure (mmHg); Delta (Δ): differences between HUT and supine values; Dis Hx: disease history (years); Edu: years of education; F: female; HR: heart rate (b/m); HUT: mean value during the last minute of head-up tilt; M: male; PAF: pure autonomic failure; Pt: patient; SBP systolic blood pressure (mmHg); Supine: mean value during the last 5 minutes of supine rest.

**Table 2:** Patients' medications.

Patient	Medications
**1**	Midodrine 2.5 mg BID; Acetylsalicylic acid 100 mg QD; Simvastatin 10 mg QD; Omeprazole 20 mg QD; Silodosin 8 mg QD; Calcitriol 0.25 QD; Captopril 25 mg QD.
**2**	Midodrine 2.5 mg BID; Fludrocortisone 0.01 mg QD; Prednisolon 25 mg QD.
**3**	Prednisolon 50 mg QD; Midodrine 2.5 TID; Domperidon 10 mg TID; Lorazepam 1 mg QD.
**4**	Droxidopa 600 mg TID; Fludrocortisone 0.01 mg QD; Tyrosine 50 mg QD.
**5**	Midodrine 2.5 mg QD; Fludrocortisone 0.01 mg QD.
**6**	Droxidopa 600 mg BID; Captopril 50 mg QD; Atorvastatin 40 mg QD
**7**	Captopril 12.5 mg QD; Nimodipin 1 drop; Lansoprazol 30 mg QD; Acetylsalicylic acid 100 mg QD; Macrogol 13.8 mg QD.
**8**	Ticlopidine 250 mg BID; Midodrine 2.5 mg BID; Fludrocortisone 0.015 mg QD; Methylprednisolone 500 mg QD; Lorazepam 2.5 mg QD; Rosuvastatin 5 mg QD.
**9**	Midodrine 2.5 mg QD; Fludrocortisone 0.01 mg QD; Nifedipine 15 drops.
**10**	Droxidopa 600 mg TID; Fludrocortisone 0.01 mg QD; Tyrosine 50 mg QD.
**11**	Lorazepam 1 mg QD; Etilephrine 7.5 mg TID; Alprazolam 0.25 mg QD.
**12**	Fludrocortisone 0.01 mg QD.

QD = once daily; BID = twice a day; TID = three times a day. Please note that patients were required to postpone their usual morning medications until after the end of the evaluation.

Patients and controls underwent neuropsychological assessment on 2 separate days in 3 different conditions: first, while seated, by means of the Brief Mental Deterioration Battery (BMDB), Word and Semantic Fluency and the Stroop Color Word Test, to evaluate global cognitive function [11]–[13]; then, the day after, by means of a selection of neuropsychological tests during supine rest (supine) and head-up tilt (HUT) to assess the effect of OH on attention and executive function. These tests were selected, on the basis of our previous experience [9], in order to reduce the time needed for this evaluation and made it feasible for our patients during the HUT. This selection included the Digit Span Forward and Backward for immediate and working memory, Barrage test for visual search function, Immediate Visual Memory for visual memory, Analogies test for verbal abstract thinking, Trail Making A and Trail Making B for attention and executive functions [13]–[16]. During HUT patients were kept to an angle ranging from 30° to 50°, able to cause a fall of at least 20 mmHg in SBP but at the same time, not to evoke symptoms. Controls were all kept to an angle of 40°.

BP and heart rate (HR) were continuously recorded, non-invasively by means of a Task Force Monitor (CNSystem, Austria) for the whole duration of the examination.

On both days participants were assessed in the morning, in a quiet clinical investigation room by the same examiner (R.P.). Healthy controls were investigated at another time than the patients and assessment of cognitive function was not blinded. Participants were required to postpone their usual morning medications until after the end of the evaluation and to abstain from smoking and drinking alcohol or caffeinated beverages from the night before the study.

Each of the HUT/supine session lasted approximately 15 min, and was separated by 30 min of supine rest. The sequence of execution of the HUT/supine evaluation was randomly assigned in order to have half of the patients and controls who performed neuropsychological assessment first during supine rest and then during HUT and the remaining half who performed the tests in the reverse order. To reduce the effect of learning, parallel forms of the tests were presented on each assessment and the sequence of presentation of the various tests was randomized. Patients' performances were compared on an individual basis to the Italian reference range values [14], [16]. The results to the neuropsychological tests during HUT and supine condition were compared by using Wilcoxon signed-ranks test for related samples. Statistical analysis was performed using IBM SPSS Statistics 20.0; a p<0.05 was considered significant.

## Results

While sitting, the Mini Mental State Examination, the final result of the BMDB (a measure of global cognition functioning), the results to Word and Semantic Fluency test and to the Stroop Color Word test were within the normal range in all patients (Table 3).

**Table 3:** Patients' results, adjusted for age and education, to the neuropsychological assessments while sitting, supine and during head-up tilt.

Patient	MMSE	FR	WF	SF	Stroop T	Stroop E	Trail Making A		Trail Making B		Trail Making B-A		Barrage		Immediate visual memory		Analogies		Span forward		Span backward	
Position	sitting	sitting	sitting	sitting	sitting	sitting	supine	HUT	supine	HUT	supine	HUT	supine	HUT	supine	HUT	supine	HUT	supine	HUT	supine	HUT
**1**	29.53	2.14	44.85	25	17.63	−1	46.5	45.5	41	−1	15	−44.5	−1.68	−0.76	22.65	20.65	18	15.2	8.25	7.25	7.23	7.23
**2**	28.31	2.47	23.6	47	7	1.25	23	33	69	84	46	51	−0.58	−0.61	21	20	19	18	6.5	5.5	5.58	5.58
**3**	28.3	2.23	17.8	37	25.5	−0.87	40	47	65	151	26	79	−0.82	0.16	17.75	14.75	15.2	**10**	8.5	5.5	5.08	4.08
**4**	27.3	2.33	33.6	38	17.75	−1.75	24	34	18	28	−5	−5	0.2	**7.13**	21	20	19.5	**14.5**	7.5	4.5	4.64	3.64
**5**	27.3	2.66	31.6	49	9.75	−1.76	27	50	19	16	−45	−33	−0.75	**3.16**	23	21	20.5	20.5	6.5	5.5	4.64	4.64
**6**	28	2.93	40.55	55	4.25	−1.37	5.5	4.5	−12	−2	−17.5	−6.5	−0.66	−1.58	22.95	21.95	20	**14**	7.25	7.25	4.33	4.33
**7**	28.4	1.78	24.9	36	19	−1.5	17	29	22	42	5	13	0.16	0.17	19.1	15.1	16	**10**	5.25	4.25	4.39	3.39
**8**	27.7	1.85	26.4	40	27.25	−1.25	29	38	−1	−3	−30	−35	−0.74	1.15	22.3	21.3	19.5	17.5	5.5	5.5	4.77	3.77
**9**	28.31	3.45	33.9	49	11.58	0.37	29.2	24.5	47.78	60	14.5	41.5	−0.73	−0.65	21.9	16.9	19	17	6.5	5.5	4.62	2.62
**10**	29.87	1.71	32.6	45	25.25	−1.37	23.5	25.5	−19.5	−12	−42.5	−36.5	−0.76	1.16	16.55	18.55	20.5	18.5	6.5	6.5	4.53	3.53
**11**	29	1.92	25.4	41	21.25	−1	30	30	−70	−16	−70	−46	2.17	**5.09**	16.3	15.3	17.5	**10.5**	5.75	5.75	3.97	3.97
**12**	28	2.53	18.2	39	15.5	−1.25	19	41	−16	28	−35	−13	−0.72	0.16	20.8	20.8	20	**12**	8.25	7.25	4.28	4.28
**N.V.**	>23.8	>0	>17.35	>24	<27.5	<7.5	<93	<93	<283	<283	<187	<187	<2.50	<2.50	>13.85	>13.85	>15.1	>15.1	>3.75	>3.75	>2.65	>2.65

FR: final result of Brief Mental Deterioration battery; HUT: head-up tilt; MMSE: Mini Mental State Examination; N.V.: normal values; SF: semantic fluency; WF: word fluency; Stroop T: time needed to perform Stroop test; Stroop E: errors performed during Stroop test. Data highlighted in bold are outside normative range according to the Italian standardization.

**Figure 1:**
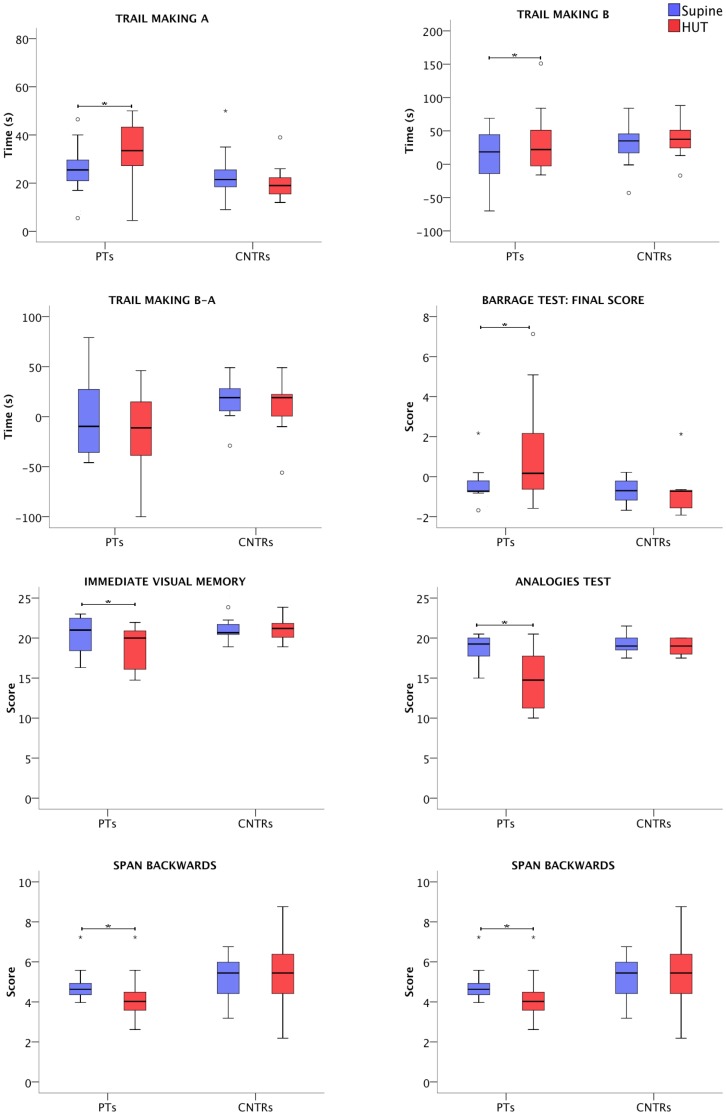
Results of the neuropsychological tests during the head-up tilt (HUT) and supine sessions (Supine) in patients (PTs) and controls (CNTRs). * = p<0.05.

Compared to supine assessment, during HUT patients scored significantly worse in the Trail Making Test A (p = 0.021) and B (p = 0.034), Barrage test final score (p = 0.013), Analogies test (p = 0.003), Immediate Visual Memory (p = 0.019), Span Forward (p = 0.008) and Span Backward test (p = 0.020) (Figure 1 and Table 3).

Despite this significant worsening of executive functions, reversible pathological scores, with regard to Italian reference range values, were observed during HUT in the Barrage test and in the Analogies test only in 3 and 6 patients respectively (Table 3).

On the contrary, controls' results to neuropsychological tests were not significantly different during HUT and supine assessment (Figure 1).

To exclude a possible learning effect of test-retest, the results obtained in the last assessment of the second day were compared to the results obtained in the previous session, irrespectively of the position in which they were performed (supine/HUT), and no statistically significant differences were observed in any of the performed test.

## Discussion

These results demonstrate that patients with peripheral AF present a significant reversible worsening of immediate and working memory, sustained attention, visual search and abstract thinking during the orthostatic challenge, but a normal global cognitive function while seated. Pathological scores may be observed in a minority of patients only during HUT. On the contrary, no changes in cognitive function were observed in healthy controls during HUT compared to supine assessment.

These data confirm our previous results and indicate that, even after a prolonged disease history, OH “per se” does not seem to be associated with permanent cognitive deficits. Our data suggest that the previously reported association between OH and cognitive decline may be the consequence of other factors, such as the presence of white matter lesions, silent cerebral infarcts or central neurodegeneration, possibly sharing the same pathogenesis of OH. On the contrary, the BP fall observed during the orthostatic challenge was associated with a reversible impairment of executive function, which may be related to systemic hypotension with transient cerebral hypoperfusion. This hypothesis is strengthened by a previous brain SPECT study, in which orthostasis caused a decreased blood flow in frontal areas in a patient with PAF, which reversed to normal values while lying flat [17].

The absence of changes in neuropsychological results in healthy controls during supine and HUT assessment indicate that our results on PAF and AAF are solid and are not affected by the motor performance related to the supine/HUT position or to a possible learning effect.

We believe this data add valuable information to the current knowledge of these rare disorders and may help to clarify previous results regarding the relationship between OH and cognitive function. Moreover, they are clinically relevant to understand that patients with OH, if needed, have to be neuropsychologically tested in supine position possibly with BP monitoring and considering all the conditions that are known to worsen OH such as food, alcohol and hypotensive drugs.
